# Geology and the Bible

**Published:** 1875-05

**Authors:** 


Geology and the Bible.—It is well known, that when the study of geology first arose, it was involved in interminable schemes of reconciliation with the letter of Scripture. There were and are two modes of reconciliation, which have each totally failed. The one attempts to wrest the words of the Bible from their real meaning, and force them to speak the language of science; and the other attempts to falsify science to meet the supposed requirements of the Bible. The "Seventy," finding that the hare was described as chewing the cud, inserted the word "not;" and on the other hand the Jesuits, in edititing Newton's "Principia," announced in the preface that they were constrained to treat the theory of gravitation as a fictitious hypothesis, else it would conflict with the decrees of the popes against the motion of the earth.

But there is another reconciliation of a higher kind, or rather not a reconciliation, but an acknowledgment of the affinity and identity which exist between the spirit of science and the spirit of the Bible. First, there is a likeness of the generl spirit of the Bible truths; and, secondly, there is a likeness in the methods.

For instance, the geological truth which our illustrious student was the chief instrument in clearly setting forth and establishing was the doctrine, wrought out by careful, cautious inquiry in all parts of the world, that the frame of this earth was brought into its present condition not by sudden and violent convulsions, but by the slow and silent action of the same causes which we see now, but operating through a long succession of ages beyond the memory and imagination of man. There need be no question whether this doctrine agrees or not with the letter of the Bible. We do not expect it would. For, had there been no such scientific conclusions, we now know perfectly well, from our increased insight into the nature and origin of the early biblical records, that they were not and could not be literal, prosaic, matter-of-fact descriptions of the beginning of the world, of which, as of its end, no man knoweth or can conceive except by figures or parables. It is now clear to all students of the Bible that the first and second chapters of Genesis contain two narratives of the creation side by side, differing from each other in almost every particular of time and place and order. It is now known that the vast epochs demanded by scientific observation are incompatible both with the 6,000 years of the Mosiac chronology and the six days of the Mosaic Creation. No one now infers from the Bible that the earth is fixed, that it cannot be moved, that the sun does literally go forth as a bridegroom from his chamber, or that the stars sung with an audible voice in the dawn of the creation.—*Dean Stanley, in the Funeral Discourse of Sir Charles Lyell, in Popular Science Monthly for May.*

